# Onset of psychiatric signs and impaired neurocognitive domains in inherited metabolic disorders: A case series

**DOI:** 10.1002/jmd2.12133

**Published:** 2021-02-09

**Authors:** François Medjkane, Marine Bohet, Marielle Ister, David Cohen, Aesa Parenti, Majda Janati, Karine Mention, Dries Dobbelaere, Renaud Jardri

**Affiliations:** ^1^ CHU Lille Service de Psychiatrie Enfants et Adolescents, Centre de Référence des Maladies Rares à Expression Psychiatrique, Hôpital Fontan Lille France; ^2^ Service de Pédiatrie Hôpital Victor Provo Roubaix France; ^3^ Département de Psychiatrie Enfants et Adolescents AP‐HP, GH Pitié‐Salpêtrière Paris France; ^4^ Institut des Systèmes Intelligents et de Robotiques CNRS UMR‐7222, UPMC, Sorbonne Universités Paris France; ^5^ Reference Centre for Inherited Metabolic Diseases in Child and Adulthood, University Children's Hospital Jeanne de Flandre and RADEME Lille Cedex France; ^6^ University of Lille, INSERM U‐1172 CHU Lille, Lille Neuroscience and Cognition Centre (LiNC), Plasticity and Subjectivity team (PSY team) Lille France; ^7^ CHU Lille Psychiatry Unit of the Clinical Investigation Centre (CIC‐1403), CURE Platform, Fontan Hospital Lille France

**Keywords:** cognition, development, DSM, psychiatry, rare disease, RDoC

## Abstract

Inherited metabolic disorders (IMDs) can present with psychiatric signs that vary widely from one disease to another. This picture is further complicated by the fact that these features occur at very different illness time points, which may further delay appropriate diagnosis and treatment. In this case series of 62 children and adolescents suffering from IMDs, we clustered psychiatric signs (on the basis of the fifth edition of the Diagnostic and Statistical Manual for Mental Disorders classification) as well as impaired cognitive domains (on the basis of the Research Domain Criteriamatrix) according to their mean age of onset (5.7 ± 4 years). We observed consistent patterns of occurrence across disorders. Externalizing symptoms, sleep problems, and cross‐domain self‐regulation deficits were found to precede the IMD diagnosis. Repetitive thoughts and behaviors as well as emotional dysregulation were found to occur around the disease onset. Finally, late‐onset features included dissociative or eating disorders, together with impaired emotion knowledge. Clinicians should specifically look for the co‐occurrence of age‐specific atypical signs, such as treatment resistance or worsening with psychotropic medication in the earliest stages and symptom fluctuation, confusion, catatonia, or isolated visual hallucinations. We believe that the combined characterizations of psychiatric signs and impaired neurocognitive domains may enable the earliest detection of IMDs and the appropriate care of these particular manifestations.

**Key Points:**

- Psychiatric signs are common in inherited metabolic disorders (IMDs) and may occur in the same age‐range as other clinical manifestations.
- Three clusters of psychiatric signs and two clusters of neurocognitive domains can be defined according to their mean age of onset.
- Warning signs to be used in liaison psychiatry should include age‐specific cognitive impairments

## 1 INTRODUCTION

Inherited metabolic disorders (IMDs) represent a vast and heterogeneous collection of genetic conditions that impact the normal functioning of systemic biochemical processes.^1^ More than 800 rare genetic diseases fit this definition. When considered in isolation, each of these IMDs is quite rare, but together, they may affect at least 3.5% to 5.9% of the worldwide population.^2^ Early detection of IMD appears to be critical as late diagnosis can lead to severe neurodevelopmental degeneration and death.^3^ In contrast, if recognized early, some IMDs may benefit from specific treatments that allow prevention or stabilization of the clinical condition; some conditions may even reach partial recovery.^4^, ^5^, ^6^ An example is phenylketonuria, which is systematically screened for at birth and treated with a specific diet to prevent neurodevelopmental consequences. It is now well known that metabolic defects can deeply impact physiological central nervous system functioning, but despite such awareness, the exact place for psychiatric assessment in diagnosis strategies remains unclear.

Because psychiatric manifestations of IMDs are wrongly considered late onset, there is a risk that first psychiatric manifestations will be overlooked. This does not mean that all of psychiatric signs reported in IMDs occur during childhood, but that most of them will and that they can even be the only symptoms for many years.^6^ Because they occur within the same age range, psychiatric manifestations of IMDs constitute an accurate phenocopy of major childhood mental disorder categories (eg, attentional deficit with or without hyperactivity, neurodevelopmental disorders including autism, mood disorders, psychosis, or anxiety), further delaying adequate care.

Interestingly, several attempts have been made to increase medical condition recognition in childhood psychopathology. First, some authors have proposed atypical signs or red flags in the context of acute psychosis, such as catatonia or cognitive regression.^7^, ^8^ Second, causality scores have been developed to better select young patients with a higher probability of medical conditions.^9^, ^10^ Third, some experts have recently emphasized the need to move from conventional categorical diagnoses to more translational domains in the characterization of rare genetic disorders in psychiatry.^11^ Such a translational approach aims to better capture the full spectrum of phenotypic severity in rare diseases and improve the understanding of their neurodevelopmental origin across disorders.

Of course, some medical aspects are crucial, and it appears to be clinically useful to group IMDs according to their onset and course: (a) clinical emergencies, characterized by an acute episode followed by recurring episodes of confusion (eg, urea cycle disorders and porphyria); (b) chronic treatable diseases (eg, Wilson disease and some lysosomal storage disorders); and (c) chronic less‐treatable diseases (eg, homocystinuria and late‐onset metachromatic leukodystrophy). However, a more dimensional approach may also help clinicians from different fields assess and manage psychiatric manifestations, even in the earliest stages of these disorders.

To investigate the potential discriminant values of both psychiatric signs and dimensions, we took advantage of the data from a series of children and adolescents with IMDs referred to the Lille University Reference Center for Inherited Metabolic Diseases (G2 MLille). To do so, we retrospectively explored medical records not only for psychiatric signs, which are known to vary widely across IMDs but also for neurocognitive trait distribution over time, both in accordance with recent nosological systems (ie, the fifth edition of the Diagnostic and Statistical Manual for Mental Disorders (DSM‐5^12^) and the *National Institute of Mental Health Research Domain Criteria* (Research Domain Criteria [RDoC]^13^). Our aim was to determine whether we could use psychiatric signs and cognitive domains to narrow the differential diagnosis for first‐line health care providers who may consult on patients with psychiatric manifestations.

## 2 METHODS

### 2.1 Participants and data extraction

We retrospectively collected data from the medical records of all children and adolescents born between 1994 and 2010 and followed‐up at the G2 MLille Center. These data were anonymously used in accordance with general data protection regulation European legislation and after collecting written consent from the patients and their parents. Patients referred to the G2 MLille Center are notably assessed using a multidisciplinary work‐up that includes neuropediatric and psychiatric assessments and a systematic psychological and neurocognitive exploration. We created a grid for data extraction that listed all the retained variables of interest (see Section 2.2). For each of these variables, we indicated their occurrence time in relation to the time of diagnosis. To ensure the validity of data extraction, two authors (M. I. and D. D.) first extracted the data from all medical records. In cases of disagreement, the other senior authors (R. J. and F. M.) were consulted.

### 2.2 Variables of interest

The first extracted variable of interest was the presence/absence of psychiatric signs according to the DSM‐5 classification during the course of the IMD (DSM criteria were revised within the study timeframe and were retrospectively applied to the medical report descriptions). Psychiatric signs could have been observed during acute or more stabilized stages of the disorders. We also collected descriptive data regarding medical history, clinical examinations, and complementary investigations, as well as the personal‐family context. We notably gathered the following data: (a) the age at the onset of the first psychiatric and nonpsychiatric signs (to specify an acute or a progressive onset as well as the primary or secondary nature of the symptoms); (b) the presence of trigger factors; (c) the presence of previously described atypical clinical features^6^ (ie, catatonia, unimodal visual hallucinations, delirium, clinical fluctuations and cognitive regression); and finally (d) the therapeutic management and response (including the propensity to experience side effects or worsening under psychotropic medications after 4 weeks of continuous treatment) and follow‐up information.

Based on systematic psychological and neurocognitive assessments, we further reported the presence/absence of a particular deficit among four constructs previously linked with developmental psychopathology^14^ based on the RDoC framework, that is, the delay of gratification (the ability to delay hedonically attractive rewards), the regulation of frustration (the ability to modulate or adjust the intensity of the affective response to frustration), executive control (the ability to deploy attention and inhibit some responses, based on Wechsler IQ scale subtests), and finally, emotion knowledge (the ability to understand emotional expressions and social contexts).

### 2.3 Statistical analyses

Statistical analyses were performed using R software for statistical computing v3.6.1, including the jmv, ggplot, and ggridges libraries. The sample was first described based on symptom frequency rates and the visualization of symptom distribution over time. Due to nonnormality, we used nonparametric tests for statistical comparisons. Continuous variables were compared using Welch's *t* tests or Wilcoxon rank tests (for independent and paired samples, respectively). Dichotomous variables were compared using chi‐squared tests. Significance was assigned to a *P* value below .05.

## 3 RESULTS

A total of 62 children and adolescents with rare IMDs were included in the series. The etiological distribution of this sample is summarized in Table 1. On average, the diagnosis of an IMD was made at 5.2 years old (SD = 4.6, ranging from birth to 18 years old). In total, 36 (58%) children and adolescents exhibited multiple psychiatric signs during follow‐up. In 11 (31.4%) of these children, psychiatric signs were present at onset, and in 15 (42.9%) patients, they even preceded the IMD diagnosis. Among the 29 patients described as having a progressive onset, 6 exhibited acute psychiatric manifestations. Regarding trigger factors, hypercatabolic episodes (ie, intense physical activity, infections) or nonobservance of the increased protein diet in patients at risk for hyperammonemia were identified. Some alerting signs that are regularly described as atypical in psychiatric disorders were also reported with a high prevalence; these included isolated visual hallucinations (17.1% vs 12.5% in the general pediatric population^25^), delirium (28.5% vs 21% on average in children hospitalized in ICU independently of IMDs), symptom fluctuations (54.3%), and inefficacy or poor tolerance of psychotropic drugs (42.9%, 9/21 who received such medication).

**TABLE 1:** Diagnostic list of the 1994‐2010 case series from the Lille University Reference Center for Inherited Metabolic Diseases, with respective gene(s), OMIM entries, frequency, and counts for psychiatric signs

Etiological Categories	Gene (inheritance): OMIM	Prevalence in the sample, n (%)	Current freq. of psych. signs, n (%)	Previously reported freq. psych. signs (%)
Urea cycle disorders	OTC (*X‐linked R*): 311 250	15 (24.2%)	8 (53.3%)	21^15^
Hyperammonaemia due to NAGS deficiency	NAGS (*AR*): 237 310	1	1 (100%)	NA
Wilson disease	ATP7B (*AR*): 277 900	2	1 (50%)	51^16^
Homocystinurias	CBS (*AR*): 236 200	5 (8.1%)	3 (60%)	64^17^
Acute intermittent porphyria	HMBS (*AD*): 176 000	2	0	30^18^
Niemann‐Pick C disease	NPC1, NPC2 (*AR*): 257 220	2	1 (50%)	40^19^
Neonatal adrenoleukodystrophy	PEX (*AR*): 202 370, 266 510, 601 539	3 (4.8%)	1 (33.3%)	NA
Propionic aciduria	PCCA, PCCB (AR): 606 054	2	0	21^20^
Fabry disease	GLA (*X‐linked R*): 301 500	5 (8.1%)	0	4^21^
X‐linked adrenoleukodystrophy	ABCD1 (*X‐linked R*): 300 100	2	1 (50%)	50^22^
Hexosaminiadase A deficiency	HEXA (*AR*): 272 800	1	0	40^22^
Alpha mannosidosis	MAN2B1 (*AR*): 248 500	1	0	25^23^
Neuronal ceroid lipofuscinoses	CLN3, CLN8 (*AR*): 204 200, 600 143	4 (6.5%)	3 (75%)	74^24^
Mucopolysaccharidosis type II	IDS (*X‐linked R*): 309 900	3 (4.8%)	3 (100%)	NA
Mucopolysaccharidosis type III	SGSH, NAGLU (*AR*): 252900,252 920	4 (6.5%)	4 (100%)	NA
Nonketotic hyperglycinemia	GLDC, AMT (*AR*): 605 899	2	2 (100%)	38^22^
Smith‐Lemli‐Opitz syndrome	DHCR7 (*AR*): 270 400	4 (6.5%)	4 (100%)	NA
MELAS syndrome	Unknown (*MA*): 540 000	4 (6.5%)	3 (75%)	NA
Total		62	36 (58%)	

*Note*: The general frequency of psychiatric signs for each disease (when available) is provided in the right column (readers should notice that we did not considered isolated *mental retardation* as a psychiatric sign).

Abbreviations: AD, autosomal dominant; AR, autosomal recessive; MI: mitochondrial inheritance; NA, not available; NAGS: N‐acetylglutamate synthase; X‐linked R, X‐linked recessive.

**FIGURE 1:**
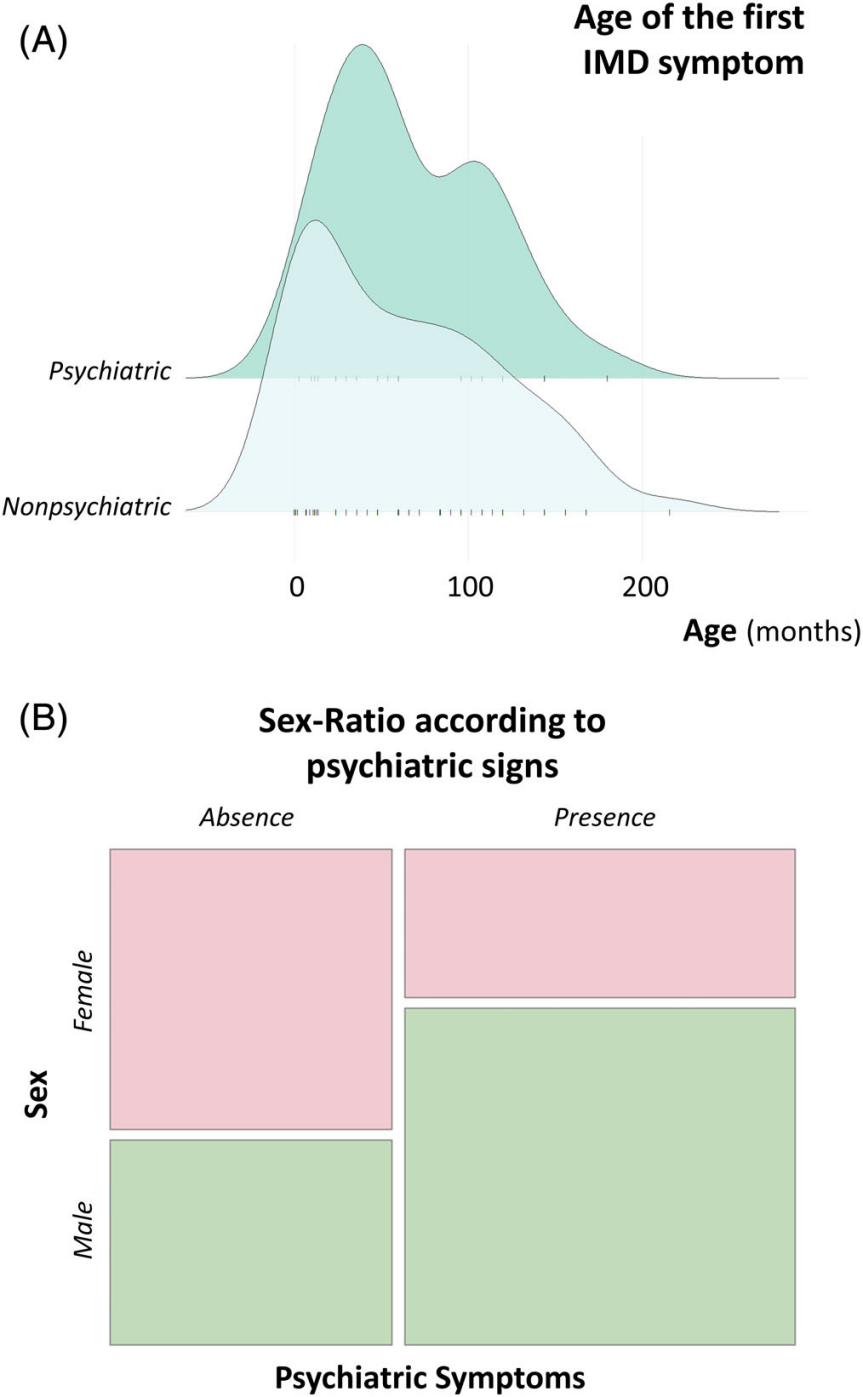
Sociodemographic of children and adolescents referred to the Lille University Reference Center for Inherited Metabolic Diseases (IMDs, n = 62). A, Comparative age distribution of the first psychiatric (dark green) and nonpsychiatric (light green) symptoms in IMD patients. The mean age of onset for psychiatric and nonpsychiatric signs in children with IMDs is similar. On average, nonpsychiatric symptoms are first diagnosed at 5.2 ± 4.6 years old, while the first psychiatric signs are reported at 5.6 ± 3.7 years old. B, Mosaic plot of sex distribution according to the presence/absence of psychiatric signs. Males with IMDs (green) exhibited psychiatric signs more often than females with IMDs did (pink, *P* = .03). This corresponds to a 2.3 male‐to‐female sex ratio for IMDs with psychiatric signs vs a 1.4 female‐to‐male sex ratio among those without such clinical features

We found no significant difference in the mean age at diagnosis between the IMD patients with or without psychiatric features in this sample (n = 62, mean age: 69.9 ± 60.3 and 52 ± 47.6 months in patients with or without psychiatric signs, respectively, *t*_60_ = −1.31, *P* = .196). Furthermore, in a more detailed examination of the subsample of IMD patients with psychiatric features, we found no significant difference between the age of occurrence of the first psychiatric symptom (5.6 ± 3.7 years old) and the age of occurrence of any other clinical manifestation (5.8 ± 5 years old; n = 36, *W* = 224, *P* = .644). The sample distribution by the age of occurrence of psychiatric and nonpsychiatric signs is reported in Figure 1A. Interestingly, a differential sex distribution was observed; there were significantly more males in the sample with psychiatric signs (male‐to‐female sex ratio = 2.3) than in the subsample without such clinical features (female‐to‐male sex ratio = 1.4; n = 62, chi‐squared = 4.57, *P* = .03, see Figure 1B).

**FIGURE 2:**
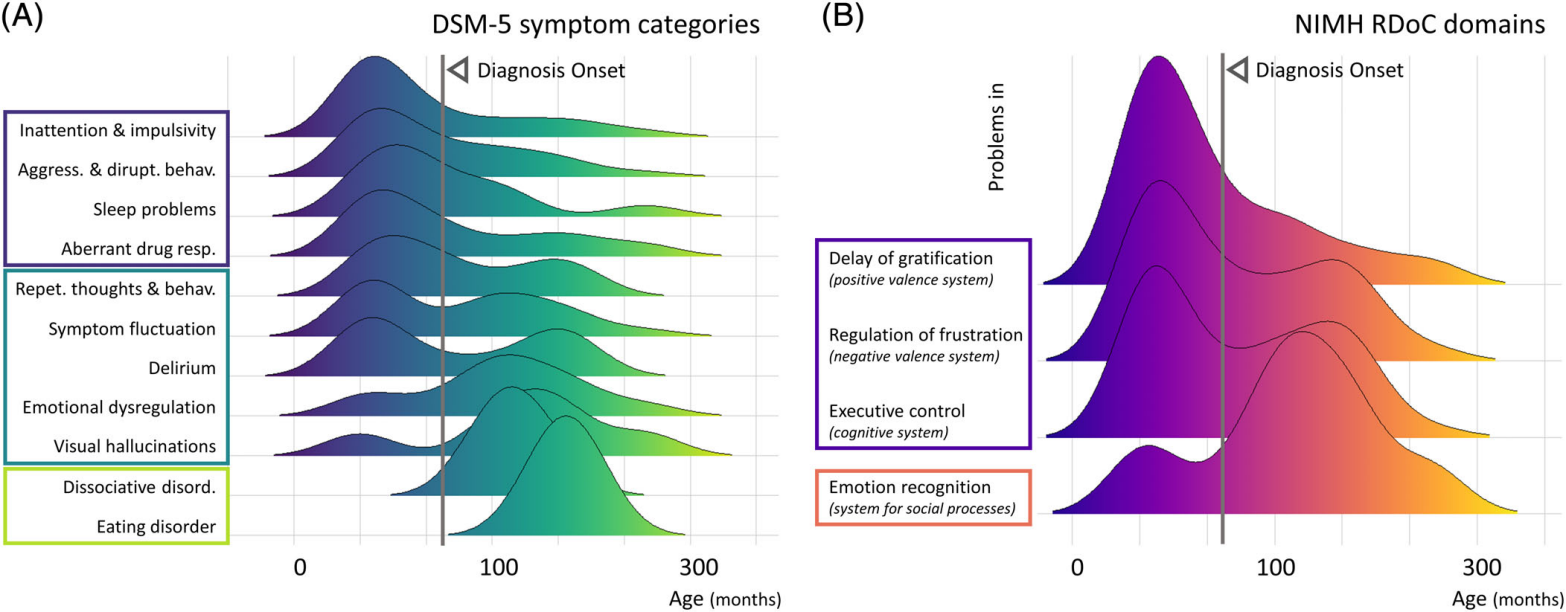
Ridgeline plots of the fifth edition of the Diagnostic and Statistical Manual for Mental Disorders (DSM‐5) symptoms and Research Domain Criteria (RDoC) in children and adolescents from the Lille University Reference Center for inherited metabolic diseases (IMDs, n = 62). A, Symptom distributions according to DSM‐5 categories are aligned to the same time scale. Among these symptoms, three clusters can be identified in regard to the mean age at IMD diagnosis (62.4 months, represented as a vertical gray line). First, framed in violet: inattention and impulsive behaviors, aggressive and disruptive behaviors, sleep problems, and aberrant drug response precede, on average, the IMD diagnosis. In contrast, dissociation and eating problems occur, on average, after the IMD diagnosis (framed in light green). A third category of symptoms framed in dark turquoise exhibits a bimodal distribution, may precede or follow diagnosis (ie, repetitive thoughts and behaviors, visual hallucinations, symptom fluctuation, delirium and emotional dysregulation). B, Domain distributions according to the RDoC matrix aligned to the same time scale. Four behavioral and cognitive constructs that have been previously proposed in the context of early childhood were used. Two clusters could be identified. First, framed in violet, self‐regulation deficits across multiple domains (frustration, executive control, and delay of gratification) occur early in the course of the disease, while problems in emotional recognition and understanding often occurred later (framed in orange), after the mean age of IMD diagnosis (vertical gray line)

Regarding the nature of psychiatric features, the IMD patients mainly presented externalized symptoms (80.5%), followed by sleep disorders (28.6%), autistic traits (25.7%), and depressive symptoms (20%). A fine‐grained exploration of the distribution of these DSM‐5 symptoms as a function of time allowed us to identify three different patterns of occurrence during the natural course of the disease (see Figure 2A). A first subset of psychiatric signs occurred on average before or at the time of the IMD diagnosis (purple cluster in Figure 2A, left). This was the case for inattention and impulsivity (4.32 years old), aggressive and disruptive behaviors (5 years old), sleep problems (5 years old), and aberrant drug response (5.7 years old). In contrast, another subset of psychiatric signs occurred late in the course of the disease; these included dissociative symptoms (9.7 years old) and eating disorders (13 years old) and are represented in the green cluster in Figure 2A, left. Finally, a third cluster of psychiatric signs exhibited two peaks of occurrence around the mean age of IMD diagnosis (blue cluster in Figure 2A, left). This was the case for repetitive thoughts and behaviors, delirium, emotional dysregulation, visual hallucinations, and symptom fluctuations.

Regarding neurocognitive domains, the IMD patients mainly exhibited problems with executive control functioning, which occurred in 35.5% (n = 22) of patients. In addition, 29% exhibited problems delaying gratification (n = 18), 25.8% had problems regulating frustration (n = 16), and 14.5% had difficulty recognizing emotion (n = 9). We applied the same visualization and analysis scheme for these RDoC constructs (Figure 2B) and identified two patterns of cognitive traits based on their distribution around the mean age at diagnosis. First, deficits in the ability to pay attention and inhibit responses to stimuli (executive control, 6.1 years old) and to delay rewards (delay of gratification, 4.5 years old), or regulate frustration (5.9 years old) occurred early in the course of the IMD (purple cluster in Figure 2B, left), mainly before or at the time of the diagnosis. In contrast, emotional knowledge was found to be impaired later in the course of the disease (9.4 years old, orange cluster in Figure 2B, left).

## 4 DISCUSSION

It has been regularly described that metabolic disorders that disrupt neurodevelopment may result in psychiatric syndromes, but this case series is the first to specifically address the profile and timing of psychiatric manifestations of IMDs according to recent nosological classifications and the major impacted neurocognitive domains.^11^, ^14^ Although these findings needs to be interpreted with caution due to the presence of X‐linked disorders in the sample (see Table 1), we first evidenced that boys with IMDs experience psychiatric signs more frequently than girls with IMDs do. For one‐third of the whole sample, these psychiatric manifestations were primary, in accordance with previous reports showing that such clinical signs may remain isolated for years, notably in Niemann‐Pick C disease,^26^ adrenoleukodystrophy,^27^ Wilson disease,^28^ or cerebrotendinous xanthomatosis.^29^ This is critical because four of these diseases do have specific therapeutic options (ie, miglustat in Niemann‐Pick C disease, haematopoietic stem cell therapy in X‐linked adrenoleukodystrophy, chelating agents in Wilson disease, and chenodeoxycholic acid replacement therapy for cerebrotendinous xanthomatosis).^7^ Because clinical symptoms stay the most directly observable features of a disease, the early co‐occurrence of psychiatric and nonpsychiatric signs in the same sage‐range (~5‐6 years old) supports the idea of a common origin (ie, linked with IMDs) for both of these markers.

Another strength of this case series is its ability to extract symptoms and domain clusters based on their time of occurrence. Using ridgeline plots, we represented psychiatric signs and cognitive trait distributions as a function of time and in reference to the age at diagnosis. A total of 83% of children with psychiatric signs also presented with neurocognitive impairments. Because the RDoC approach allows the results to be organized on the basis of putative mechanisms, we believe that this partial co‐occurrence with psychiatric features illustrates the complementarity of the two approaches. For instance, beyond a strict acute/chronic distinction, we were able provide the first evidence regarding one extreme of the spectrum: externalizing symptoms (ie, problems with the self‐control of emotions and behavior), sleep problems and cross‐domain self‐regulation deficits, which often preceded the IMD diagnosis. Though poorly specific taken in isolation, the co‐occurrence of these signs should warn the clinician, notably when they are associated with resistance or worsening in response to psychotropic medication. On the other extreme of the spectrum, late‐onset features encompass psychotic dissociative or eating disorders together with emotional recognition and understanding, which occur on average several years after the IMD diagnosis. Around the time of diagnosis, the most frequent psychiatric features are repetitive thoughts and behaviors and emotional dysregulation. Again, this analysis showed that clinicians should specifically look for the co‐occurrence of period‐specific atypical signs, such as symptom fluctuation, delirium or isolated visual hallucinations, for which the prevalence was found to be higher than in clinical and nonclinical populations.

We need to acknowledge some limitations of the work. First, the data set is heterogeneous since we collapsed markedly different IMD manifestations and typical ages of presentation, making the generation of disease subtype‐specific recommendations impossible. Second, the absence of a difference in the mean age of onset between the first psychiatric signs and other clinical manifestations should be interpreted with caution. However, the similarity of their respective distributions (see Figure 1A) supports the idea that psychiatric signs occur on average within the same age range as other features. Second, despite their clinical relevance and all the efforts made to exhaustively collect the necessary data from the medical records of the retained 62 children with IMDs and properly assign DSM‐5 symptoms and RDoC traits, the outcome measures were defined a posteriori to patient enrollment, exposing this case series to potential reporting biases.

We cannot exclude the possibility that such bias may account for the absence of notified catatonia in this sample, despite previous descriptions of this alerting sign in the literature.^8^ This may also be a result of the nonsystematic use, until recently,^30^ of adapted rating scales for catatonia in children, such as the pediatric catatonia rating scale (PCRS).^31^ Of note, other first‐line warning signs reported in the literature^4^, ^6^, ^18^ were correctly identified and extracted from the sample. As previously stated, we were able to move beyond what was previously known and identify early and late patterns of occurrence for these atypical signs.

Overall, we confirmed that psychiatric manifestations are frequent and highly polymorphic in IMD and occur within a range of occurrence similar to that indicated in isolated previous reports (Table 1). By providing evidence of clusters of symptoms and cognitive domains with specific onset profiles (ie, before, at same age as and after the IMD diagnosis), we hope that such findings, if replicated, could pave the way for the development of more global and interdisciplinary management algorithms for children suffering from IMDs. First‐line clinicians should consider using neurocognitive domain clusters as useful tools to complement psychiatric red flags in children with (or at risk of) IMD due to the high co‐occurrence of these signs during specific disease stages. A better detection of associated cognitive traits as a complement to the categorical diagnostic procedure appears to be crucial from a global health perspective not only for early detection and appropriate care but also for screening various levels of risk and for informing family planning and support.

## ETHICS APPROVAL AND CONSENT TO PARTICIPATE

This study received a CNIL‐DEC2015‐152 authorization. Written consent was obtained from each patient and their parents.

## CONSENT FOR PUBLICATION

Not applicable.

## CONFLICT OF INTEREST

The authors declare no potential conflict of interest.

## AUTHOR CONTRIBUTIONS

Marielle Ister and Dries Dobbelaere collected the data. François Medjkane and Renaud Jardri performed the first analyses. All the authors were involved in discussing the findings and writing the manuscript. They all approved its final version.

## DATA AVAILABILITY STATEMENT

The anonymized data set is available from the corresponding author upon reasonable request.
